# Reprogramming Human Female Adipose Mesenchymal Stem Cells into Primordial Germ Cell-Like Cells

**DOI:** 10.1007/s12015-023-10561-x

**Published:** 2023-06-20

**Authors:** Giulia Salvatore, Susanna Dolci, Antonella Camaioni, Francesca Gioia Klinger, Massimo De Felici

**Affiliations:** 1 Department of Biomedicine and Prevention, University of Rome Tor Vergata, Via Montpellier 1, Rome, 00133 Italy; 2 Saint Camillus International University Of Health Sciences, Via di Sant’Alessandro 8, Rome, 00131 Italy

**Keywords:** Primordial germ cells, Artificial gametes, Adipose-derived mesenchymal stromal cells, Human infertility, PGCLCs

## Abstract

In the last two decades, considerable progress has been made in the derivation of mammalian germ cells from pluripotent stem cells such as Embryonic Stem Cells (ESCs) and induced Pluripotent Stem Cells (iPSCs). The pluripotent stem cells are generally first induced into pre-gastrulating endoderm/mesoderm-like status and then specified into putative primordial germ cells (PGCs) termed PGC-like cells (PGCLCs) which possess the potential to generate oocytes and sperms. Adipose-derived mesenchymal stromal cells (ASCs) are multipotent cells, having the capacity to differentiate into cell types such as adipocytes, osteocytes and chondrocytes. Since no information is available about the capability of female human ASCs (hASCs) to generate PGCLCs, we compared protocols to produce such cells from hASCs themselves or from hASC-derived iPSCs. The results showed that, providing pre-induction into a peri-gastrulating endoderm/mesoderm-like status, hASCs can generate PGCLCs. This process, however, shows a lower efficiency than when hASC-derived iPSCs are used as starting cells. Although hASCs possess multipotency and express mesodermal genes, direct induction into PGCLCs resulted less efficient.

**Supplementary Information:**

The online version contains supplementary material available at 10.1007/s12015-023-10561-x.

## Introduction

Adipose tissues contain cells of mesenchymal origin termed adipose-derived mesenchymal stromal cells (ASCs). ASCs are proliferative and multipotent cells, having the capacity to differentiate mainly into cell types such as adipocytes, osteocytes and chondrocytes. The use of adipose tissue is advantageous for regenerative medicine because it is an easy-to-harvest and abundant source of ASCs. Additionally ASCs can be efficiently isolated, maintained and propagated in vitro [1].

The multipotency of ASCs can be upgraded to pluripotency by introducing the four canonical reprogramming transcription factors, OCT4, SOX2, KLF4 and c-MYC. The resultant adipose-derived induced pluripotent stem cells (iPSCs) exhibit nearly all the characteristics and morphologies of embryonic stem cells (ESCs) from the corresponding species. Mouse and human ASCs are over 5-fold and 100-fold more efficiently reprogrammable into iPSCs than fibroblasts, which are usually the most commonly used cell types for iPSC generation [2].

In the last two decades, considerable progress has been made in the derivation of mammalian germ cells from ESCs and iPSCs, which are regarded as a suitable experimental model for elucidating mammalian germ cell development and potential strategies for producing haploid germ cell in vitro.

In all mammalian embryos, the earliest identifiable germ cells are primordial germ cells (PGCs), which are specified in extraembryonic regions during pre/early-gastrulation stages. Following complex developmental processes that include genome wide reprogramming, migration from the yolk sac and proliferation, PGCs arrive inside the developing testes or ovaries where they differentiate into prospermatogonia or oogonia/oocytes, respectively, from which spermatogenesis or oogenesis move forward. In the last twenty years, as mentioned above, on the basis of the identification of the transcription factors guiding PGC specification mainly in the mouse embryo, this process was reproduced in vitro from both mouse and human ESCs and iPSCs. The pluripotent cells are generally first induced into pre-gastrulating epiblast-like cells (EpiLCs) and then specified into PGC-like cells (PGCLCs), which possess the developmental capability for both spermatogenesis and oogenesis (for a review, see [3]). In the mouse, PGCLCs obtained from ESCs or iPSCs were then matured into eggs able to generate healthy pups both in vivo and in vitro [4, 5].

Protocols for generating human PGCLCs (hPGCLCs) from ESCs and iPSCs result in the formation of cells equivalent to migratory PGCs at around week 3 of embryo development, also termed early PGCs [6, 7]. Such cells were more recently further differentiated into pre-meiotic oogonia-like cells through aggregation with mouse embryonic ovarian somatic cells [8]. Alternatively, another study has demonstrated that human female ESCs can directly differentiate into VASA-positive oocyte-like cells by transduction of the mRNA binding proteins DAZL and BOULE [9].

hPGCLCs were also generated from Multilineage-differentiating Stress Enduring (Muse) cells obtained from amniotic membrane [10]. Muse cells are a small population of pluripotent stem cells, originally isolated from bone marrow aspirates and human skin fibroblasts but also found in ASCs (for a review, see [11]).

Male mouse ASCs, dedifferentiated in vitro into ESC-like cells by the four canonical reprogramming factors, have been reported to form embryonic bodies (EBs) which can be induced to differentiate into epiblast-like cells (EpiLCs) by activin A and, subsequently, into colonies of PGCLCs by BMP4. Such PGCLCs expressed SSEA-1, BLIMP-1, and STELLA and showed initial epigenetics changes typical of embryonic PGCs [12]. Male mouse ASCs have also been directly differentiated into PGCLCs using media containing BMP4 [13] or retinoic acid (RA) [14] or both compounds in sequence [15]. Under these two latter conditions, the authors claimed to have obtained PGCLCs able to differentiate further into prospermatogonia/gonocytes. Similarly, male canine ASCs, differentiated into EBs or grown in monolayers, were induced into PGCLCs and putative gonocytes by BMP4 [16] or overexpression of CD61 (integrin-β3) via TGF-β [17]. Finally, human male ASCs treated with BMP4 or transfected with miR-106b gave rise to PGCLCs [18] or more advanced germ-like cells expressing *OCT4*, *PIWIL2*, *ITGB1*, *SSEA-1* and *STRA8*, following 21 days of culture in the presence of RA [19].

Since no information is available about the capability of female hASCs to be reprogrammed into PGCLCs, we used and compared protocols to produce such cells from hASC-derived iPSCs or directly from hASCs themselves.

## Materials and Methods

### hASC Isolation and Culture

hASCs were obtained following a previously published protocol [20]. Briefly, cells obtained by lipoaspirate digestion were cultured in αMEM (Aurogene) supplemented with 10% FBS (Gibco), 2 mM L-glutamine, 100 UI/ml penicillin and 0.1 mg/ml streptomycin (all from Sigma-Aldrich) at 37 °C and 5% CO_2_, with culture medium changes every 2–3 days. After reaching approximately 80% confluency, cells were passaged by 5 min incubation with Trypsin-EDTA (Sigma-Aldrich) and seeded at concentration of 1000 cells/cm^2^. In all experiments, cells were used at passage 3–5. Their identity as ASCs was confirmed through cytofluorimetric analysis and differentiation potential, as previously described [20]. Lipoaspirate samples were collected from adult healthy women, after signed informed consent under the authorization number 160/20 from the Ethical Committee of Fondazione PTV Policlinico Tor Vergata.

### hiPSCs Culture

hiPSCs were cultured in Essential 8™ Medium (Gibco) in feeder-free condition in 6-well plates coated with Geltrex (Geltrex™ LDEV-Free Reduced Growth Factor Basement Membrane Matrix, Gibco) according to the manufacturer’s protocol. Colonies were passaged in clumps every 4–5 days, when they reached approximately 85% confluency, by incubation for 3 min with an EDTA solution consisting in 0.5 mM EDTA (Sigma-Aldrich) in PBS (Sigma Aldrich) supplemented with 0.18% NaCl (VWR). If single-cell passaging was needed instead, cells were pre-treated for 1 h with 10 µM Rocki (STEMCELL Technologies) before being detached with the EDTA solution as above for 10 min. When plated as single cells, 10 µM Rocki was added for the first 18–24 h of culture. To cryopreserve hiPSCs, PSC Cryomedium (Gibco) was used. At thawing, RevitaCell™ Supplement (Gibco) was added for the first 18–24 h of culture.

### hiPSCs and hASCs Induction into PGCLCs

For hiPSC differentiation into Primordial Germ Cells-Like Cells (PGCLCs), the protocol by Sasaki et al. [7], with minor modifications was used. Briefly, to induce hiPSCs into iMeLCs (incipient Mesoderm-Like Cells), 6 × 10^4^ cells/cm^2^ were plated onto Geltrex-coated plates (Geltrex™ LDEV-Free Reduced Growth Factor Basement Membrane Matrix, Gibco) in GK15 medium (GMEM medium containing 15% KSR (Gibco), 0.1 mM NEAA, 2 mM L-glutamine, 1 mM sodium pyruvate (Sigma Aldrich), and 0.1 mM 2-mercaptoethanol (Gibco), additioned with 50 ng/ml Activin A, 3 µM CHIR99021 (Sigma-Aldrich), and 10 µM ROCKi (STEMCELL Technologies). After 48 h, cells were detached with Accumax solution (Invitrogen) and induced into hPGCLCs for 6–9 days by plating 3 × 10^3^ cells/well into a V-bottom 96-well plate with cell repellent surface (Greiner Bio-One) in 100 µl of GK15 supplemented with 100 ng/ml LIF (corresponding to 1000 U/ml), 200 ng/ml BMP4, 100 ng/ml SCF, 50 ng/ml EGF (all purchased from Peprotech), and 10 µM ROCKi (STEMCELL Technologies). In order to differentiate hASCs into hPGCLCs, the same two step protocol was used, with the difference that for iMeLCs induction only 1.2 × 10^4^ cells/cm^2^ were plated, or, alternatively, direct induction of hASCs into hPGCLCs, without iMeLCs pre-induction, was attempted.

### Total RNA Extraction and cDNA Synthesis

Total RNA was extracted from cells using TRIzol reagent (Invitrogen). Passages were performed according to manufacturer’s instructions and the extracted RNA was quantified with NanoDrop (ND-1000 Spectrophotometer, Thermo Fisher Scientific). The first strand cDNA was synthesized from RNA template by QuantiTect Reverse Transcription kit (QIAGEN).

### RT-qPCR

Real Time Polymerase Chain Reaction (RT-qPCR) were set up with SsoAdvanced™ Universal SYBR® Green Supermix (Bio-Rad) and PrimePCR™ SYBR® Green Assays (Bio-Rad). Amplification was performed on a Light Cycler 96 (Roche). The thermal cycling conditions were 95 °C for 30 s, 40 cycles of 95 °C for 15 s and 60 °C for 60 s, followed by melting curves. Data from the reaction were collected and analyzed using the comparative 2^− ΔΔCt^ method. Relative quantification of gene expression was performed relating the signal in the induced samples to that of the untreated control. Gene expression was normalized to PPIA (Peptidylprolyl Isomerase A) and PrimePCR™ Control Assay were used to evaluate Reverse Transcription performance (qHsaCtlD0001001), RNA quality (qHsaCtlD0001002), RT-qPCR performance (qHsaCtlD0001003) and gDNA contamination (qHsaCtlD0001004). Unsupervised hierarchical clustering (UHCA) and Principal component analysis (PCA) (explaining 95% variance) was performed with Orange software. Primers used are reported in Table S1.

### Immunofluorescence

For immunofluorescence on EB-like cell aggregates obtained from iPSCs whole mount was used following the protocol by Hikabe et al. [5] with slight modifications. Briefly, cell aggregates were fixed in 2% PFA for 20 min and after three washes in PBST2 (PBS containing 0.2% Triton X-100), blocked in PBS containing 0.1% BSA and 0.3% Triton X-100 overnight at 4 °C. Then, samples were incubated overnight at 4 °C with the first primary antibody (goat anti-SOX17) in blocking buffer. The next day, aggregates were washed five times with PBST2, and were incubated with the first secondary antibody (anti-goat) for 4 h at 4 °C in blocking buffer. After five washes in PBST2, samples were incubated overnight at 4 °C with the last two primary antibody (mouse anti-PRDM1 and rabbit anti-AP2G) in blocking buffer. Finally, after five washes in PBST2, EB-like cell aggregates were incubated with the last two secondary antibody (anti-mouse and anti-rabbit) together with Hoechst for 4 h at 4 °C in blocking buffer, washed five times in PBST, and mounted in PBS-glycerol (1:1).

In order to increase the staining resolution, in ASC-derived aggregates, a different fixation and permeabilization protocol, according to Redondo-Castro et al. [21], was used. Briefly, cell aggregates were fixed in 2% PFA for 20 min and after two 15 min washes in PBST1 (PBS containing 0.1% Triton X-100), dehydrated in increasing methanol solutions (10%, 20%, 50%, 75% and 95% in PBS) for 15 min each at 4 °C, and then in 100% methanol overnight at 4 °C. The next day, samples were rehydrated with the same methanol solutions in decreasing order and then blocked in PBS containing 3% BSA and 0.1% Triton X-100 overnight at 4 °C. Incubation with the first primary antibody (goat anti-SOX17) was carried out in blocking buffer overnight at 4 °C. The next day, after three 30 min washes with PBST1, aggregates were incubated with the first secondary antibody (anti-goat) for 4 h at 4 °C in PBST1 and then, after three 30 min washes in PBST, with the other two primary antibodies (mouse anti-PRDM1 and rabbit anti-AP2G) overnight at 4 °C in blocking buffer. On the last day, after three washes in PBST1, aggregates were incubated with the last two secondary antibody (anti-mouse and anti-rabbit) and Hoechst for 4 h at 4 °C in PBST1, washed 3 times in PBST1, and mounted in PBS-glycerol (1:1). The aggregates were imaged with Nikon AX confocal microscopy, denoised, and deconvolved with NIS-Elements software. To count the number of double and triple positive cells the best-focused plane, containing at least 200 cells for each aggregate, was selected. For each of four independent experiments, at each time-point, the percentages of positive cells from three aggregates for each cell line were averaged. The list of antibodies used is reported in Table S2.

### Statistical Analysis

Data collected were analyzed with GraphPad Prism (software version 7.0, San Diego, CA). Results were given as mean ± SD and *P* value was determined by One-Way Anova and Bonferroni post-analyses or t-test. Statistical significance was based on *P* value: p < 0.05, p < 0.01, p < 0.001 and p < 0.0001 are indicated with * (or a), ** (or b), *** (or c) and **** (or d), respectively.

## Results and Discussion

### hiPSC and hASC Induction into PGCLCs: Gene Expression Analysis of Differentiation States

Female hASCs, isolated and propagated in vitro as previously described [20], were reprogrammed into iPSCs under feeder-free condition by nucleoporation with episomal plasmids as reported in Supplementary Methods (Fig. S1).

To verify the possibility to generate female PGCLCs from the hASC-derived iPSCs or directly from hASCs, we used the protocol described by Sasaki et al. [7], as detailed in M&M.

**Fig. 1:**
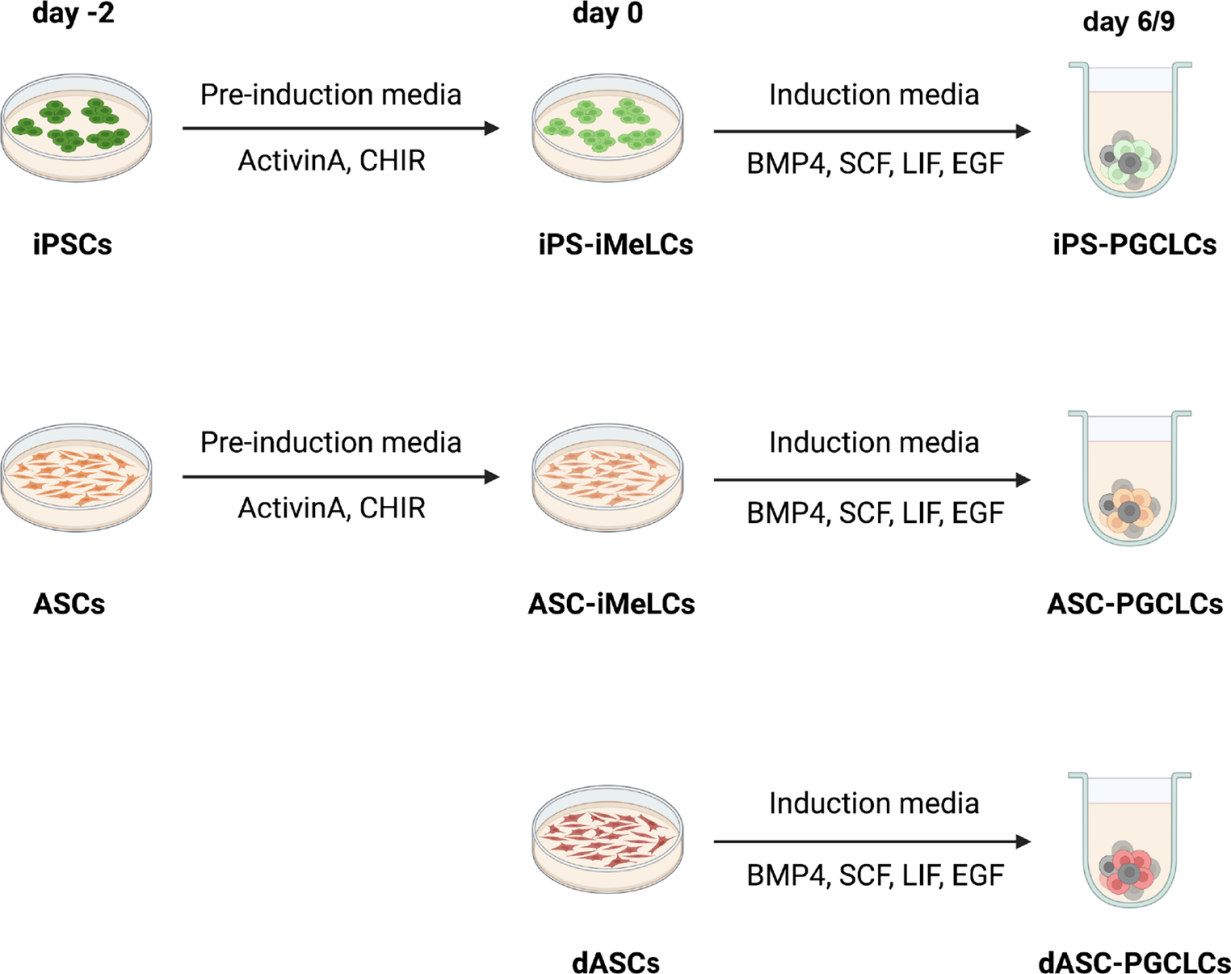
Schematic representation of the protocols used to generate hPGCLCs from hiPSCs and from hASCs

Briefly, iPSCs and ASCs were cultured in the pre-induction medium containing Activin A and CHIR for 2 days, in order to establish in these cells the peri-gastrulating endoderm/mesoderm-like status thought to be a prerequisite for PGC specification [7]; we termed such cells iPS-iMeLCs and ASC-iMeLCs (incipient Mesoderm-Like Cells derived from iPSCs and ASCs, respectively). EB-like cell aggregates were then generated by culturing such cells into low-attachment 96-well plates in the PGCLC induction medium containing BMP4, SCF, EGF and LIF for 6–9 days (obtaining iPS-PGCLCs and ASC-PGCLCs). We also produced EB-like cell aggregates directly from ASCs (dASCs) without previous exposure to the pre-induction medium, and, thereafter, cultured the aggregates in the same PGCLC induction medium described above to produce PGCLCs (obtaining dASC-PGCLCs) (Fig. 1).

**Fig. 2:**
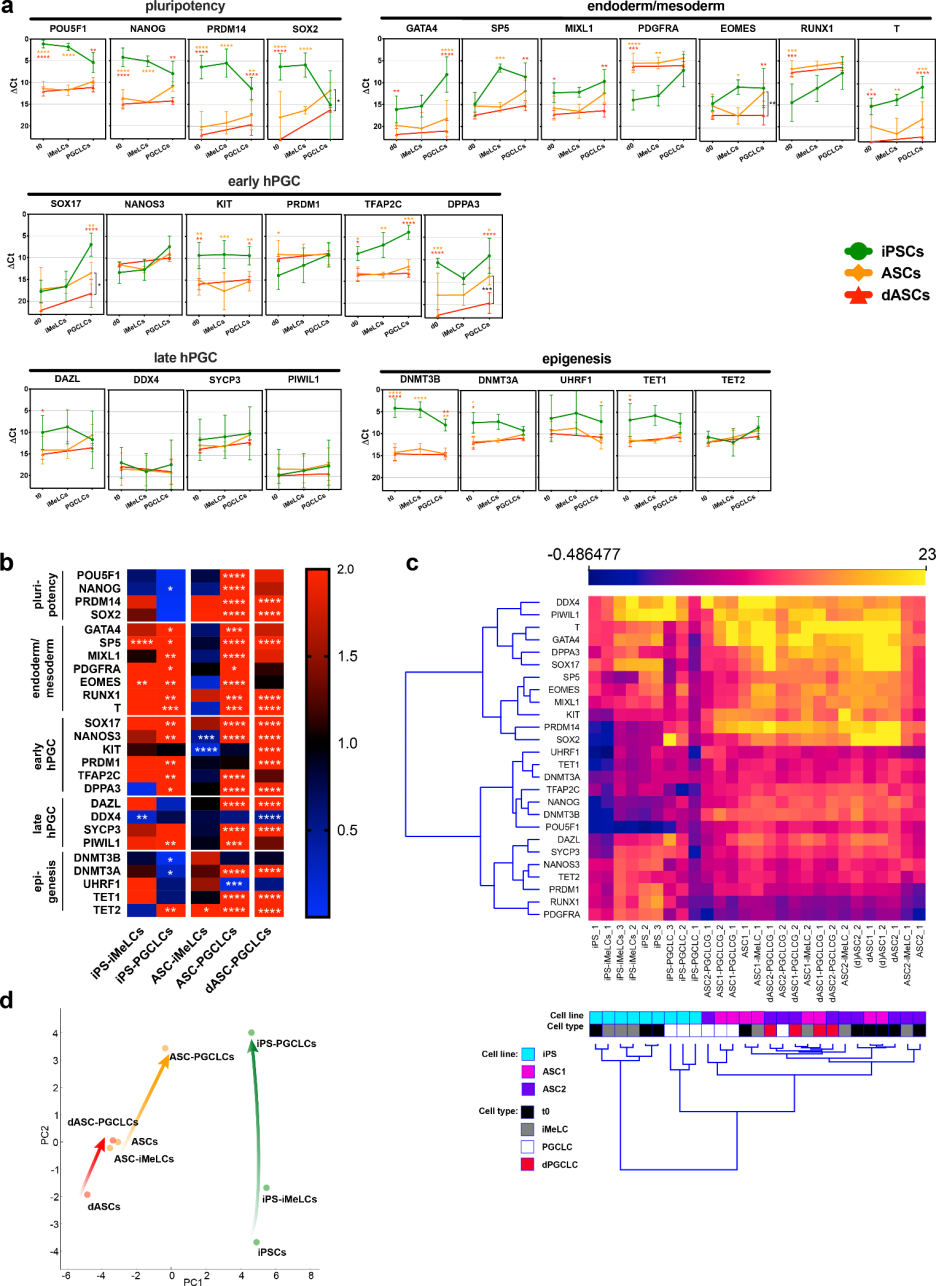
Variation of ΔCt obtained from qRT-PCR performed on hiPSCs and hASC during the different protocols used to generate PGCLCs. **a**: Time course of ΔCt values during the induction protocol. Values are expressed as mean±SD of three (iPSCs) or four (dASCs) independent experiments. Statistical significance is calculated vs. the corresponding iPS-derived cell population for each time point. Significance is colour coded as follows: orange asterisks = iPSCs vs. ASCs, red asterisks = iPSCs vs. dASCs, black asterisks = ASCs vs. dASCs. **b**: Heat map showing the mean fold change, resulting from three (iPSCs) or four (dASCs) independent experiments at each protocol steps. Fold change and statistical significance are calculated vs. the respective t0 for each sample with One Way ANOVA (iPSCs and ASCs) or t test (dASCs) and shown only when RQ≤0.5 or RQ≥2. Bright red is for RQ≥2. **c**: Unsupervised hierarchical clustering based on ΔCt value of each experiment. **d**: PCA analysis of the mean ΔCt values for each cell line and protocol. Arrows indicate differentiation trajectories. Peptidylprolyl Isomerase A (PPIA) was used as housekeeping gene. ASC1: donor 1; ASC2: donor 2

To evaluate the effects of the induction protocols on iPSCs and ASCs, we performed a detailed qRT-PCR analyses on the EB-like cell aggregates to detect the expression of various classes of genes known to be involved in hPGC specification. ΔCt values obtained from qRT-PCR performed on iPSCs and ASCs at t0 and throughout the PGC induction protocols including six days PGCLC induction are shown in Fig. 2a, while the quantitative evaluation of the qRT-PCR relative to t0 about all classes of the analysed genes are shown as heatmap in Fig. 2b. Of note, the results reported here were obtained from one iPSC line and from ASCs obtained from two female donors, which gave similar outcomes (at least two qRT-PCRs/status per each ASC line) (Fig. S2).

#### Stemness Gene Expression

In iPSCs, the transcripts of the pluripotency genes *POU5F1*, *NANOG*, *PRDM14* and *SOX2* showed similar pattern from being abundant at t0 to little change after pre-induction (iMeLCs) followed by decrease after 6-day PGC induction (PGCLCs), especially evident for *PRDM14* and *SOX2*. At t0, in ASCs the amount of *POU5F1* and *NANOG* transcripts was significantly lower and increased throughout inductions at level comparable to iPS-PGCLCs in ASC-PGCLCs but not in dASC-PGCs. The levels of *PRDM14* and *SOX2* transcripts were even lower at t0 and showed a progressive increase both in ASC-PGCLCs and dASC-PGCs; however, only for *SOX2* the amount of mRNA became comparable to that relatively low of iPS-PGCLCs.

Considering these first results, we can observe that, as expected, the levels of mRNAs of all analysed pluripotency genes were high in iPSCs at t0 and showed a tendency to decrease throughout the PGC induction protocol. Notably, at t0, appreciable basal amounts of *POU5F1* and *NANOG* transcripts were also present in ASCs. Such levels showed a tendency to increase, together with those much less abundant of *PRDM14* and *SOX2*, throughout the PGC induction almost matching in ASC-PGCLCs those of iPS-PGCLCs, except for *PRDM14*. Differently, in dASCs, such increase did not occur *(POU5F1* and *NANOG*) or occurred at levels significantly lower than in iPS-PGCLCs (*PRDM14*) or ASC-PGCLCs (*SOX2*). Of note, *PRDM14* has been reported to be expressed at low levels and *SOX2* repressed in hPGCLCs [22, 23].

#### Endo/mesodermal Expression Signature

At t0, the amounts of mRNAs of the most part of the endodermal/mesodermal genes analysed namely *GATA4*, *SP5*, *MIXL1 EOMES* and *T*, were moderate and similar in iPSCs and ASCs whereas those of *PDGFRA* and *RUNX1* were significantly higher in ASCs than in iPSCs. In these latter, the levels of *SP5* and *EOMES* transcripts increased significantly following pre-induction while those of *GATA4*, *MIXL1*, *PDGFRA*, *RUNX1* and *T* rose significantly only following PGCLC induction. In ASCs, none of gene transcripts increased following pre-induction. *SP5* and *MIXL1* were present, however, at similar amounts in the putative PGCLCs generated both from iPSCs and ASCs, and at a significantly lesser extent in those generated from dASCs. mRNA of *EOMES*, a gene whose expression was reported to be especially important for hPGC specification [22], followed a similar pattern in ASC-PGCLCs but not in dASC-PGCLCs. In these latter, it appeared not upregulated remaining at levels significantly lower than those of iPS-PGCLCs and ASC-PGCLCs (p < 0.01). *GATA4* and *T* mRNAs persisted at levels significantly lower either in ASC-PGCLCs and dASC-PGCLCs in comparison to iPS-PGCLCs. As matter of fact, only these two genes (*GATA4*, *T*) were significantly higher in iPS-PGCLCs vs. ASC-PGCLCs whereas the other, except *PDGFRA* and *RUNX1*, resulted higher in *iPS-PGCLCs* than in *dASC-PGCLCs*.

Together, the results reported above confirm that in iPSCs pre-induction by Activin A and CHIR efficiently increased the transcription of crucial endodermal-mesodermal genes such as *SP5* and *EOMES.* On the other end, such increase did not occur in ASCs, probably because of their mesodermal origin. Interestingly, in such cells, the pre-induction protocol appeared to especially favour the increase of mRNAs of *SP5*, *MIXL1* and *EOMES* after PGCLC induction. The amounts of the transcripts of these genes, however, remained low in dASC-PGCLCs, suggesting an inefficient activation in these latter of the transcriptional program driving PGC specification [22].

#### PGC Expression Signature: Early and Late Genes

At t0, in iPSCs, all early hPGC gene transcripts considered in our analysis, except those of *SOX17*, were already expressed at substantial/high levels. Those of *NANOS3*, *PRDM1* (also known as *BLIMP1*), *TFAP2C*, *DPPA3* (also known as *STELLA*) and mainly of *SOX17*, appeared to progressively increase throughout inductions, whereas *KIT* mRNAs remained unaltered. In ASCs, *NANOS3* and *SOX17* transcripts, expressed at t0 at considerable and low levels, respectively, like in iPSCs, and those for *TFAP2C* and *DPPA3*, detected at lower levels, underwent a progressive increase showing in ASC-PGCLCs for *NANOS3*, but not for *SOX17, TFAP2C* and *DPPA3*, amounts comparable to iPS-PGCLCs. Differently, in ASC-PGCLCs, mRNAs of *PRDM1* and *KIT* which at t0 showed higher and lower levels than iPSCs, respectively, did not present significant changes.

It is to be noted, however, that in an independent series of time-course experiments carried out to better follow the pattern of gene expression at 2, 4, 6 and 9 days of PGCLC induction, by analysing RQ values (Fig. S3a), it appeared that ASC needed from 2 to 4 more days to significantly upregulate crucial PGC competence genes, such as SOX17 and TFAP2C, compared to iPSCs. In addition, Principal Component Analysis (PCA) plot of the mean ΔCt values showed that the ASC-PGCLCs were clearly closer to iPS-PGCLCs after 9 rather than 6 days of induction (Fig. S3b). Thus again suggesting the necessity of longer PGC induction period in such cells.

In dASCs, the transcripts of all early hPGC genes, except those of *TFAP2C* that remained unchanged, showed patterns like those of induced ASCs. Nevertheless, in dASC throughout induction the amounts of *SOX17* and *DPPA3* mRNAs remained significantly lower than in induced ASCs (p < 0.05 and p < 0.001, respectively).

Summarizing these results, we can observe that transcripts of early PGC genes were present at substantial levels in iPSCs and that the inductions increased the mRNA amounts of *NANOS3*, *PRDM1*, *TFAP2C*, maximally of *SOX17* and, to a lesser extent, of DPPA3 at levels probably necessary for PGC specification. Notably, *NANOS3* and *PRDM1* transcripts were found at basal considerable/high amounts also in ASCs while those of *SOX17*, *TFAP2C* and *DPPA3*, likely the most crucial for hPGC specification [22, 23], were significantly upregulated after double inductions. Despite dASC-PGLCs expressed high level of mRNAs for *NANOS3* and *PRDM1* they showed amounts of *SOX17* and *DPPA3* transcripts significantly lower of either iPS-PGCLCs or ASC-PGCLCs, and no upregulation of *TFAP2C.* Thus again supporting a defective PGC specification in dASCs.

In iPSCs, amongst the transcripts of the late hPGC genes, expressed at t0 at considerable (*DAZL*, *SYCP3*) or low (*DDX4*, *PIWIL1*) levels, only those of *PIWIL1* appeared to increase throughout the induction towards PGCLCs. On the other hand, in dASCs, with the exception of DAZL whose mRNA level at t0 was lower than in iPSCs, the amounts of the other transcripts remained at levels comparable to those in iPSCs despite significant increase (*DAZL* and *SYCP3* in both, *PIWYL1* in ASC-PGCLCs only*)* or decrease (*DDX4* in dASC-PGCLCs) throughout induction.

These last results support the consolidated notion that robust expression of late hPGC genes, especially those involved in the beginning of meiosis, require conditions or factors provided by the ovarian environment that are only partly reproduced by current inductive protocols [4, 8].

#### Epigenetic Remodelling Signature

At t0, the mRNAs of major genes encoding enzymes with epigenetics functions such as *UHRF1* and *TET2* were expressed in both iPSCs and ASCs at comparable substantial and moderate levels, respectively. Differently, the levels of *DNMT3A, TET1* and *DNMT3B* mRNA were significantly higher in iPSCs than in dASCs. In iPSCs, the transcripts of all these genes except *TET2* showed a tendency to decrease throughout inductions but only for *DNMT3B* and *DNMT3A* the reduction resulted significant in iPS-PGCLCs. Conversely, *TET2* mRNA underwent a significant increase during the inductions. Both in ASCs and dASCs, the amount of *DNMT3A*, *TET1* and *TET2* transcripts increased significantly during inductions remaining, however, at levels comparable to those of iPS-PGCLs. Conversely, the transcripts of *DNMT3B* were unaltered by the inductions while those of *UHRF1* showed a tendency to decrease in dASCs and significantly decreased in double induced ASCs, where they reached levels significantly lower than in iPS-PGCLCs.

These last results indicate that both iPSCs and ASCs are well equipped with transcripts of the main epigenetics players. In line with this, mRNAs encoding for DNMT1, its co-factor UHRF1 and the *de novo* methyltransferases DNMT3A, DNMT3B and DNMT3L are all expressed in early hPGCs which have completed the phase I DNA demethylation, although proteins are below the limit of detection. Furthermore, TET1 and TET2 are also expressed, with TET1 mRNA increasing in late PGCs [24]. Therefore, the trend to decrease of *DNMT3B* and *UHRF1* and conversely to increase of *TET1 and TET2* mRNAs throughout inductions found in the present analyses is in line with the genome reprogramming occurring in PGCs [24].

Unsupervised hierarchical clustering analysis based on ΔCt values of the data reported in Fig. 2c, showed that the samples obtained from double induction of iPSCs and ASCs (excluding ASC2-PGCLC_2 sample) formed separate clusters, adjacent to each other. On the other hand, dASC-PGCLC samples appeared scattered among dASCs and ASC-iMeLCs ones. Likewise, plotting the mean ΔCt values of these data using the PCA method showed that the induction trajectory of ASCs towards putative PGCs was clearly closer to that of iPSCs in comparison to that of dASCs (Fig. 2d and S2c). Thus, confirming the analyses above, this suggests a lower efficiency of the direct induction protocol to generate putative PGCs from ASCS.

### Co-expression of hPGCLC Markers in EB-cell Aggregates

**Fig. 3:**
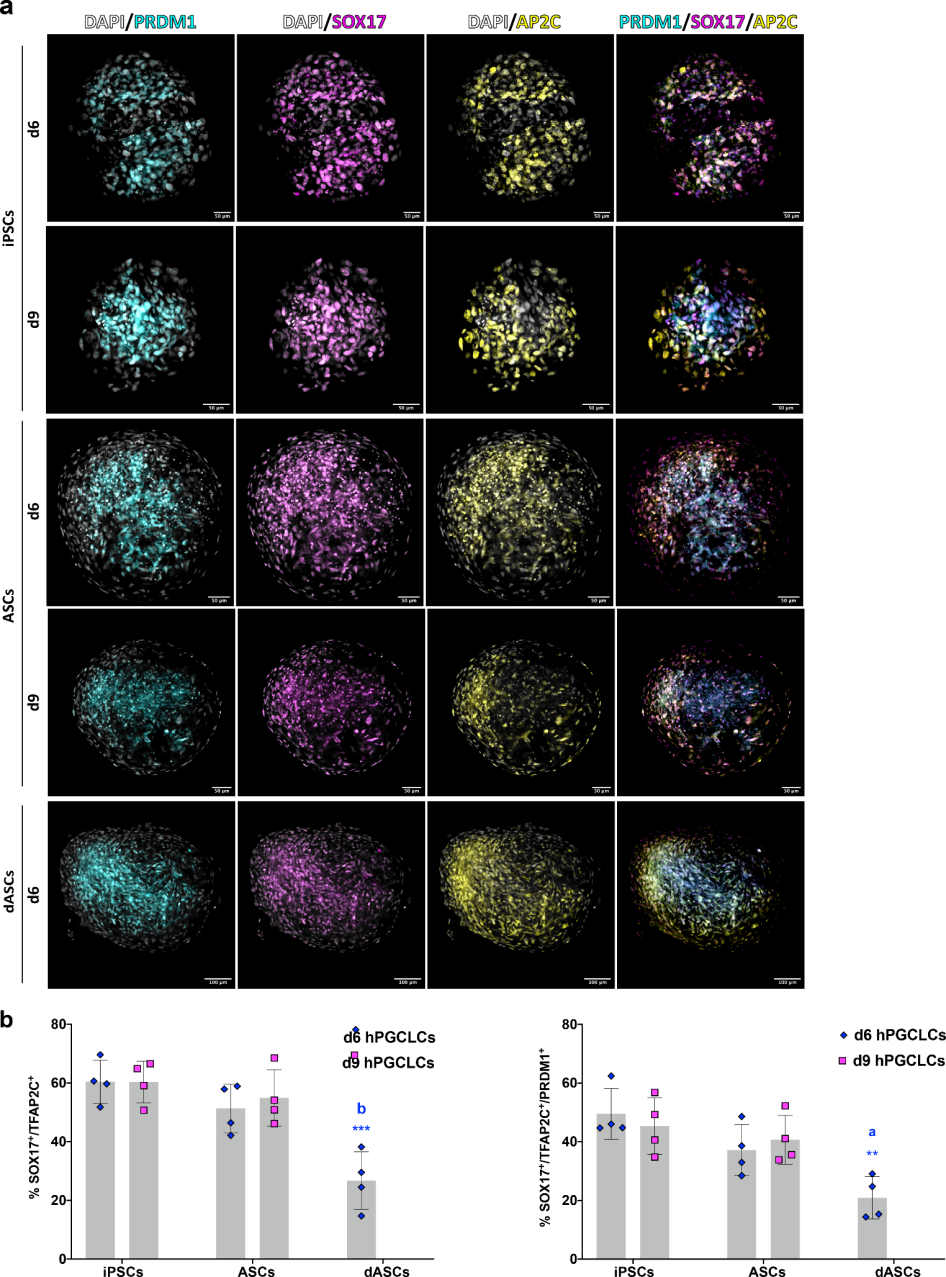
Whole mount immunofluorescence for early hPGC markers in EB-like cell aggregates. **a**: Whole mount IF in EB-like cell aggregates for markers of early PGCs PRDM1, SOX17 and TFAP2C (here AP2C) in hPGCLCs obtained from hiPSCs and hASCs following the protocols represented in Fig. 1. **b**: Percentages of cells (putative PGCLCs) double positive for SOX17 and TFAP2C (top) and triple positive for SOX17, TFAP2C and PRDM1 (bottom). Asterisk: statistical significance vs. d6 iPS-PGCLCs; letters: statistical significance vs. d6 ASC-PGCLCs

qRT-PCR results were validated by whole mount IF experiments for the early hPGC marker proteins. Staining for SOX17 and TFAP2C on the EB-like cell aggregates indicated that, after 6 days of PGCLC induction, those obtained from iPSCs consistently contained higher numbers of double positive cells than those obtained from ASCs (mean percentage ± SD; 60.43 ± 7.30 vs. 51.33 ± 8.34, n = 4); such difference was, however, not statistically significant. On the other hand, the percentage of double positive cells in EB-like cell aggregates from dASCs (26.73 ± 9.81, n = 4), was significantly lower either than iPSCs (p < 0.001) and ASCs (p < 0.01) (Fig. 3b). Likewise, the percentage of triple positive cells (PRDM1^+^/SOX17^+^/TFAP2C^+^), was higher, but not statistically significant, in EB-like cell aggregates from iPSCs compared to those from ASCs (49.49 ± 8.65 vs. 37.16 ± 8.68, n = 4), whereas EB-like cell aggregates from dASC contained significantly less triple positive cells than both the former (20.89 ± 7.20, p < 0.01 or p < 0.05 respectively, n = 4). Similar results were obtained prolonging the PGCLC induction up to 9 days, except for a slight increase of both double and triple positive cells observed in EB-like cell aggregates from ASCs (54.91 ± 9.62 and 40.68 ± 8.31 respectively, n = 4) (Fig. 3a-b), suggesting the necessity of longer PGC induction period in such cells. As reported in the previous section, the results described here were obtained from one iPSC line and ASCs derived from two female donors which gave similar outcomes (Fig. S4).

In conclusion, the IF results confirmed the qRT-analyses showing that reprogramming hASC in iPSCs is likely the best way to generate hPGCLCs. Alternatively, hASCs themselves can be used although with minor efficiency, providing pre-induction into the peri-gastrulating endoderm/mesoderm-like status is performed. On the other hand, despite hASCs possess multipotency and already express mesodermal genes, direct induction into PGCLCs appeared to be less efficient. It is likely that in such cells, a well-established mesodermal status and the lack of the genome plasticity typical of pluripotency limits such a possibility. The possibility that the PGCLCs obtained were derived from a small population (less than 9%) of pluripotent Muse cells maybe present in the hASCs [25] was not investigated in the present work, but it is worthy of consideration in future studies.

### Electronic Supplementary Material

Below is the link to the electronic supplementary material.

Supplementary Material 1. This file is still in correction mode, with the changes after revision still in red.  Moreover, in this affiliation Of is wrong. 2. Saint Camillus International University of Health Sciences, Via di Sant’Alessandro 8, 00131 Rome, Italy . Last thing to be changed is this: Correspondence email address is wrong: francesca.klinger@unicamillus.org

## Data Availability

The datasets generated during and/or analysed during the current study are available from the corresponding author on reasonable request.
