# Supplementary material for: Reprogramming Human Female Adipose Mesenchymal Stem Cells into Primordial Germ Cell-Like Cells
Source: Stem Cell Rev Rep. 2023 Jun 20;19(7):2274–83. doi: 10.1007/s12015-023-10561-x (PMC10579115; doi:10.1007/s12015-023-10561-x)
Supplement: Supplementary file 1 — Supplementary Material 1. This file is still in correction mode, with the changes after revision still in red. Moreover, in this affiliation Of is wrong. 2. Saint Camillus International University of Health Sciences, Via di Sant’Alessandro 8, 00131 Rome, Italy . Last thing to be changed is this: Correspondence email address is wrong: francesca.klinger@unicamillus.org [file 12015_2023_10561_MOESM1_ESM.docx]

**Stem Cell Reviews and Reports**

**Supplementary Information for:**

**Reprogramming human female adipose mesenchymal stem cells into primordial germ cell like cells**

Giulia Salvatore^1^, Susanna Dolci^1^, Antonella Camaioni^1^ Francesca Gioia Klinger^2^ and Massimo De Felici^1^

1. Department of Biomedicine and Prevention, University of Rome Tor Vergata, Via Montpellier 1, 00133, Rome, Italy.
2. Saint Camillus International University of Health Sciences, Via di Sant’Alessandro 8, 00131 Rome, Italy

Correspondence: defelici@uniroma2.it; klinger@uniroma2.it

**SUPPLEMENTARY TABLES**

**Table S1** List of primers used for RT-qPCR

| **Gene Name** | **UniqueAssayID** |  | **Gene Name** | **UniqueAssayID** |
| --- | --- | --- | --- | --- |
| **POU5F1** | qHsaCED0038334 |  | **EOMES** | qHsaCED0034787 |
| **NANOG** | qHsaCED0043394 |  | **RUNX1** | qHsaCID0037818 |
| **PRDM14** | qHsaCID0018592 |  | **T** | qHsaCID0011593 |
| **SOX2** | qHsaCED0036871 |  | **DAZL** | qHsaCED0042658 |
| **NANOS3** | qHsaCED0047914 |  | **VASA/DDX4** | qHsaCID0008844 |
| **KIT** | qHsaCID0008692 |  | **SYCP3** | qHsaCED0044910 |
| **BLIMP1/PRDM1** | qHsaCID0036320 |  | **PIWIL1** | qHsaCID0017852 |
| **TFAP2C** | qHsaCED0042570 |  | **DNMT3B** | qHsaCID0016111 |
| **STELLA/DPPA3** | qHsaCED0038968 |  | **DNMT3A** | qHsaCID0010863 |
| **SOX17** | qHsaCED0046246 |  | **UHRF1** | qHsaCID0009839 |
| **GATA4** | qHsaCID0012121 |  | **TET1** | qHsaCID0007976 |
| **SP5** | qHsaCED0036976 |  | **TET2** | qHsaCID0014693 |
| **MIXL1** | qHsaCED0036901 |  | **PPIA** | qHsaCED0038620 |
| **PDGFRA** | qHsaCID0007202 |  |  |  |

| **Antibody** | **Company** | **Dilution** |
| --- | --- | --- |
| NANOG (sc-293121) | Santa Cruz Biotechnology | 1:100 |
| OCT4 (sc-5279) | Santa Cruz Biotechnology | 1:100 |
| SOX2 (sc-365823) | Santa Cruz Biotechnology | 1:100 |
| SSEA-4 (sc-21704) | Santa Cruz Biotechnology | 1:100 |
| TRA-1-60 (sc-21705) | Santa Cruz Biotechnology | 1:100 |
| PRDM1 (MAB36081) | R&D | 1:60 |
| SOX17 (AF1924) | Abcam | 1:300 |
| AP2G (sc-8977) | Santa Cruz Biotechnology | 1:100 |
| Cy3-labeled anti-goat (705-165-147) | Jackson ImmunoResearch | 1:400 |
| Alexa Fluor 488-labeled anti-mouse (A-11001) | Thermo Fisher Scientific | 1:500 |
| Alexa Fluor 568-labeled anti-mouse (A-11004) | Thermo Fisher Scientific | 1:500 |
| Alexa Fluor 488-labeled anti-rabbit (A-11008) | Thermo Fisher Scientific | 1:500 |
| Alexa Fluor 568-labeled anti-rabbit (A-11036) | Thermo Fisher Scientific | 1:500 |
| Alexa Fluor 647-labeled anti-rabbit (A-21244) | Thermo Fisher Scientific | 1:400 |

**Table S2** List of primary and secondary antibodies and dilution used for IF.

**SUPPLEMENTARY METHODS**

***hASCs reprogramming into iPSCs***

hASCs were reprogrammed into human induced Pluripotent Stem Cells (hiPSCs) following a modified protocol kindly provided by Dr. Kwan Hau (Imperial College London). Briefly 6 x 10^5^  hASCs were nucleoporated with 1 μg each of the three episomal plasmids pCXLE-hOCT3/4-shp53-F, pCXLE-hSK and pCXLE-hUL (Addgene 27077, 27078, 27080) using program A-033 of the Amaxa Nucleofector I device (Lonza). After nucleofection, cells were plated on 0.1% gelatin and cultured for 6 days in normal hASCs medium. Starting from day 2, cultures were supplemented with 0.5 mM histone deacetylase inhibitor Sodium Butyrate (NaB) (STEMCELL Technologies), with daily media changes. On day 7, cells were transferred onto Geltrex-coated plates (Geltrex™ LDEV-Free Reduced Growth Factor Basement Membrane Matrix, Gibco), and on day 8 the medium was switched to Essential 8™ Medium (Gibco). NaB was added only until day 13. At day 15, colonies of putative iPSCs started to be recognizable throughout the plate and, from day 20 onward, colonies of the appropriate size (70-80% coverage of a 10x microscope field), were mechanically picked and individually cultured in feeder-free conditions. Fully reprogrammed colonies were then identified through morphological analysis (flat colonies, with clear borders and showing tightly packed cells with high nuclear-to-cytoplasmic ratio) and IF/HC for stem cell markers (see below) to confirm dim nuclear Hoechst staining and positivity for ALP, SOX2, OCT4, NANOG, SSEA-4 and TRA-1-60.

***Immunofluorescence***

For immunofluorescence (IF) on adherent iPSCs, after medium removal, cells were washed two-three times in PBS and fixed with 4% paraformaldehyde (PFA) for 10 min at RT. Cells were then permeabilized with PBS-0.01% Triton X for 10 min at room temperature (RT) and blocked in 3% BSA in PBS for 30 min at RT before incubation with primary antibodies (in 0.3% BSA in PBS) overnight at 4 °C. For double staining, samples were incubated the following day with the second primary antibody for 1 h at RT. After three 5 min washes, primary antibody binding was detected using fluorescent dye conjugated secondary antibody (1 h at RT). Hoechst was used to stain nuclei. Cells were mounted in PBS-glycerol (1:1) and observed under Leica DMI6000B microscope.

*Alkaline Phosphatase staining*

For histochemical localization of alkaline phosphatase (ALP), cells were fixed with 4% PFA for 10 min at room temperature. After three washes in PBS cells were incubated with a solution 0,01% Naphthol in 1 mg/ml of Fast Red (Sigma-Aldrich) until reaction occurred (around 15 min). Staining was analysed under a phase contrast microscope (Leitz) equipped with a DS-Fi1 camera (Nikon).

**SUPPLEMENTARY FIGURES**


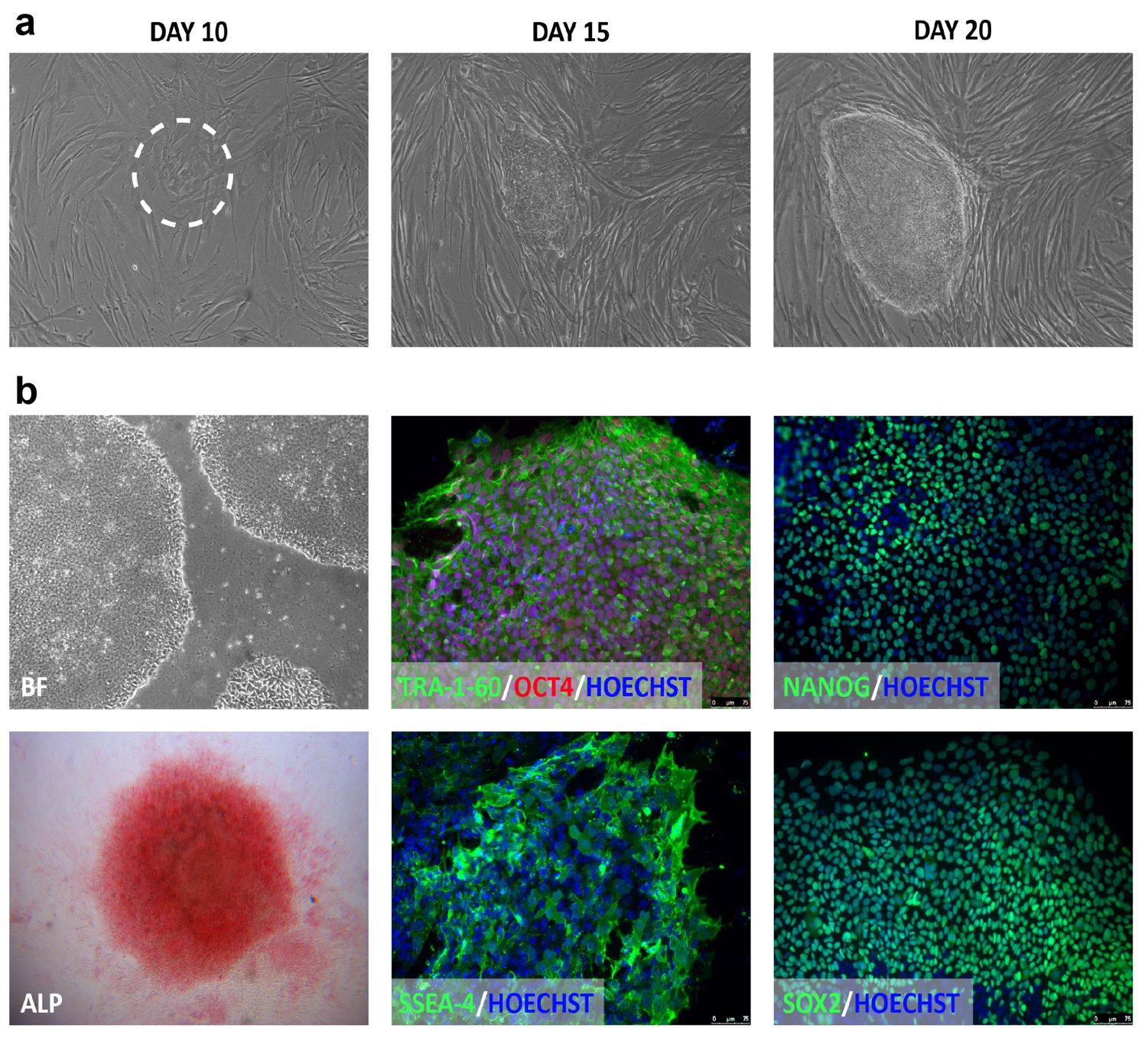


**Fig. S1 Formation of hiPSC colonies obtained from hASCs.**

**a:** Micrographs showing the time points of one iPSC colony formation. Starting at day 10 after transfection, some clusters of smaller sized cells, indicative of mesenchymal-to-epithelial (MET) transition (dotted circle), appeared in the culture. **b:** Colonies analysed after expansion in feeder-free condition: top left micrograph shows typical morphology of reprogrammed colonies (flat with clear borders, cells tightly packed with high nuclear-to-cytoplasmic ratio and prominent nucleoli); bottom left micrograph shows histochemical staining for ALP; middle and right columns show IF analysis for pluripotent stem cells markers. Such colonies were obtained with an efficiency of around 0.02%


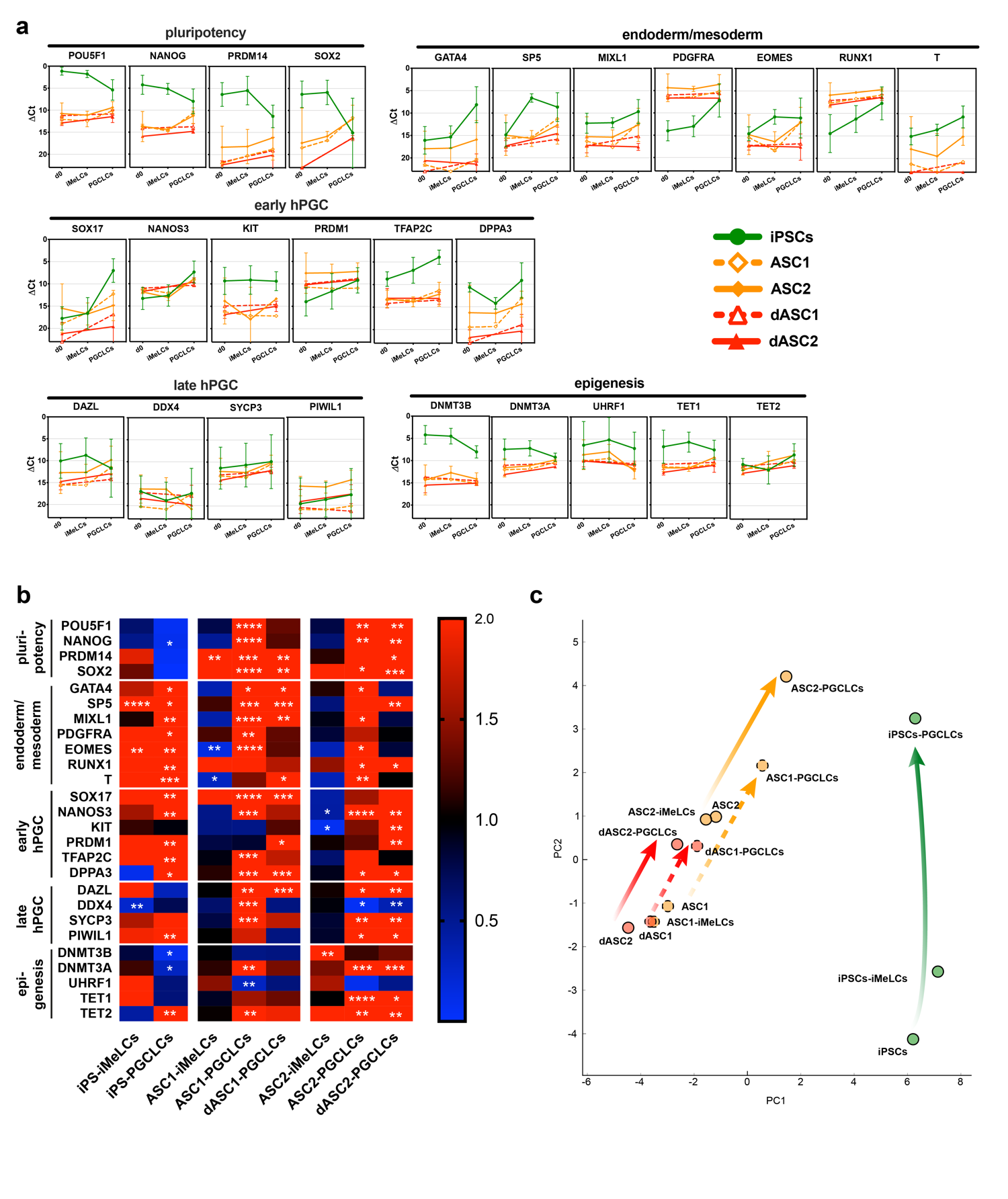


**Fig. S2 Same ΔCt values of Figure 2A during the induction protocol reported by donor.**

**a:** ΔCt values are expressed as mean±SD of three (iPSCs) or two (for each dASCs) independent experiments. Statistical significance vs the corresponding iPS-derived cells for each time point is not represented here; at each time, significance was calculated for ASC1 vs ASC2 and dASC1 vs dASC2. PPIA was used as the housekeeping. **b:** Heat map showing the mean fold change resulting from three (iPSCs) or two (for each dASCs) independent experiments at each protocol steps. Fold change and statistical significance are calculated vs the respective t0 for each sample with One Way ANOVA (iPSCs and ASCs) or t test (dASCs) and shown only when RQ≤0.5 or RQ≥2. Bright red is for RQ≥2. **c:** PCA analysis of the mean ΔCt values divided by donor. Arrows indicate differentiation trajectories


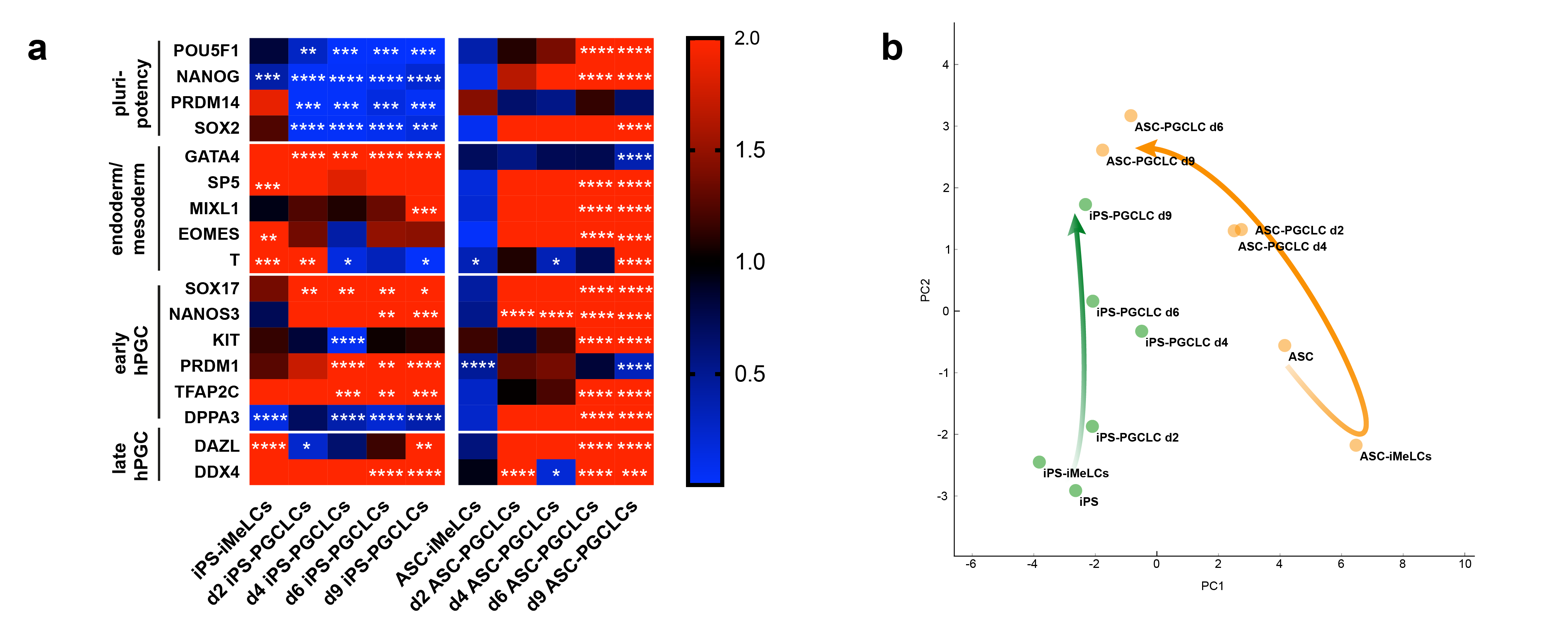


**Fig. S3 Time course experiments of gene expression at day 2, 4, 6 and 9 of PGCLC induction.**

**a:** Heat map showing the mean fold change resulting from two (iPSCs) or four (ASCs) independent time course experiments at each protocol step and time points. Fold change and statistical significance are calculated vs the respective t0 for each sample with One-Way ANOVA and shown only when RQ≤0.5 or RQ≥2. Bright red is for RQ≥2. PPIA was used as the housekeeping gene. **b:** PCA analysis of the mean ΔCt values for each cell line. Arrows indicate differentiation trajectories


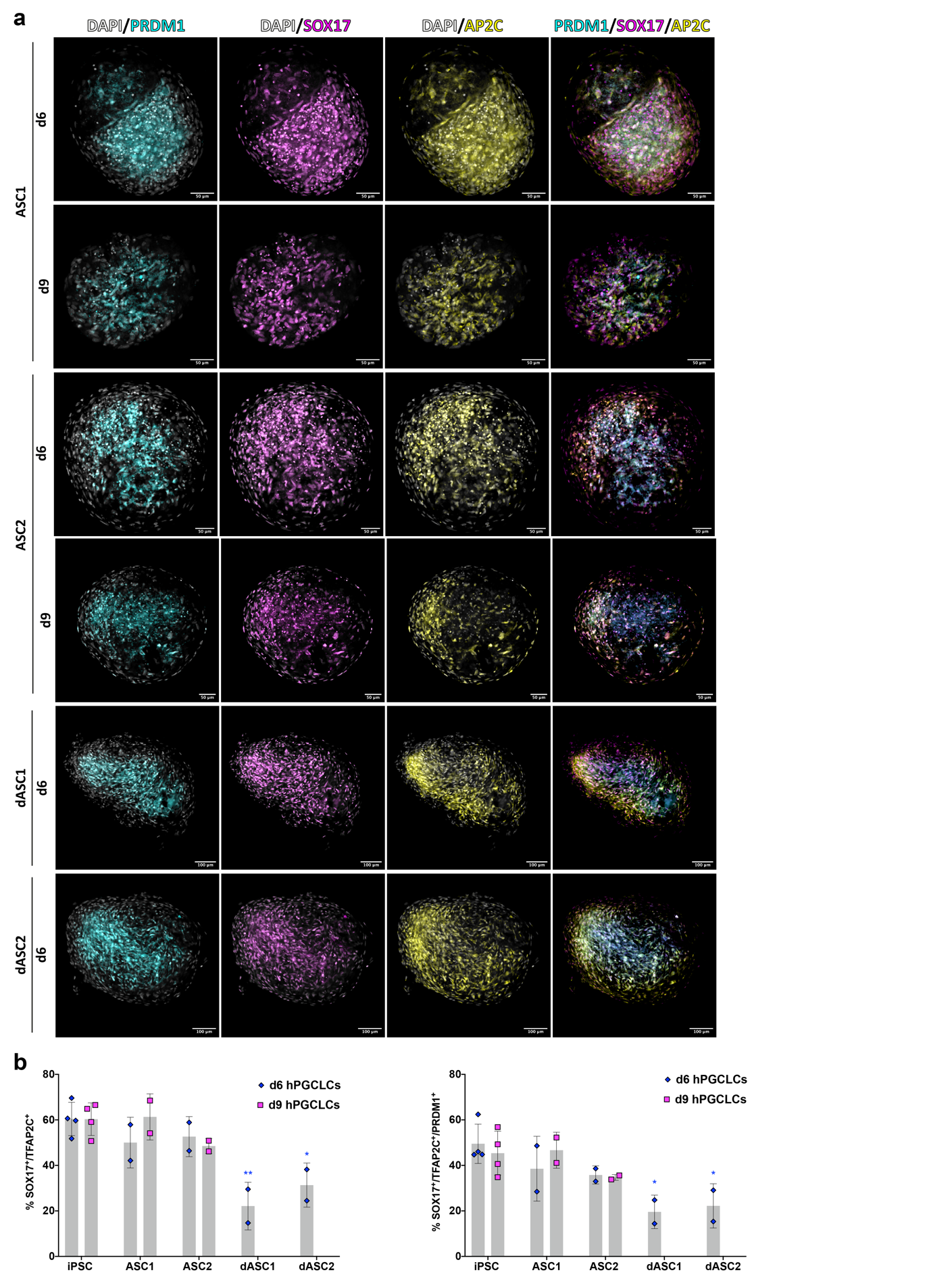


**Fig. S4 Whole mount immunofluorescence for early hPGC markers in EB-like cell aggregates obtained from ASCs of each donor.**

**a:** Whole mount IF for the early PGC markers PRDM1, SOX17 and TFAP2C (here AP2C) in EB-like cell aggregates obtained from each hASC donor following the protocols reported in figure 1. **b**: Percentages of cells (putative PGCLCs) double positive for SOX17 and TFAP2C (left) and triple positive for SOX17, TFAP2C and PRDM1 (right)
